# Effects of age on sugammadex reversal of neuromuscular blockade induced by rocuronium in Chinese children: a prospective pilot trial

**DOI:** 10.1186/s12871-021-01465-9

**Published:** 2021-10-19

**Authors:** Ruidong Zhang, Jie Hu, Shengde Li, Bin Xue, Lu Wang, Jie Bai, Jijian Zheng

**Affiliations:** 1 Department of Anesthesiology, Shanghai Children’s Medical Center, School of Medicine, Shanghai Jiaotong University, Shanghai, China; 2 Department of Anesthesiology, Qingdao Women and Children’s Hospital, Qingdao, Shandong China

**Keywords:** Children, Neuromuscular blockade, Residual neuromuscular blockade, Sugammadex, Train-of-four

## Abstract

**Background:**

Sugammadex reverses neuromuscular blockade induced by steroidal relaxants. We compared the recovery for neuromuscular blockade reversal with sugammadex in children aged 1–12 years.

**Methods:**

From August 2019 to August 2020, patients who received 2.0 mg·kg^− 1^ sugammadex for neuromuscular blockade reversal after surgery were recruited. The primary outcome was the time for the train-of-four ratio (TOFR) to recover to 0.9; secondary outcomes included the incidence of the TOFR < 0.9, extubation time, length of stay at the post-anesthesia care unit, and adverse events. Hemodynamic parameters before and 5 min after sugammadex administration and vital signs in the recovery room were also recorded.

**Results:**

Eighty-six children were recruited (1 to < 3 years, *n* = 23; 3 to < 5 years, *n* = 33; 5 to ≤12 years, *n* = 30). Intergroup differences in the recovery of the TOFR to 0.9 were not statistically significant (F = 0.691, *p* = 0.504). Recurrence of the TOFR < 0.9 was not observed in any group. Five minutes after sugammadex administration, the heart rates of patients aged 3 to < 5 and 5 to ≤12 years were significantly lower than those at baseline (*p* < 0.05). Extubation time was similar in patients aged 1 to ≤12 years. Length of stay and end-tidal capnography at the post-anesthesia care unit as well as adverse events did not differ significantly.

**Conclusion:**

A moderate (TOF count two) rocuronium-induced neuromuscular blockade can be effectively and similarly reversed with sugammadex 2 mg·kg^− 1^ in Chinese children aged 1–12 years.

**Trial registration:**

Chinese Clinical Trial Registry: ChiCTR1900023715 (June 8, 2019).

## Background

Neuromuscular blocking agents (NMBAs) are used in clinical practice to facilitate tracheal intubation, artificial ventilation, and surgical procedures [1]. If anesthesia management is based solely on clinical observation and experience, residual neuromuscular blockade (RNMB), defined as a train-of-four ratio (TOFR) < 0.9, frequently occurs. RNMB can adversely affect respiratory function and airway-protective reflexes, making patients susceptible to respiratory depression and apnea, ultimately leading to death [2, 3]. Although the incidence of RNMB related to nondepolarizing muscle relaxants is higher in adults than in pediatric patients (45.0% vs. 28.1%) [4, 5], the severity of RNMB in pediatric patients may be higher due to a lack of effective communication, monitoring, and compensation. Appropriate monitoring and complete recovery from muscle relaxation are therefore vital for safe recovery, especially in pediatric patients.

RNMB is traditionally reversed by acetylcholinesterase inhibitors, such as neostigmine. Anticholinergic agents are administered simultaneously to reduce their side effects [6]. However, this method of reversing muscle relaxation takes a relatively long time, and subsequent recurarization can occur [3]. A chemical modification of γ-cyclodextrin, named sugammadex, was recently introduced [7]. It can encapsulate rocuronium molecules with high affinity to reverse muscle relaxation induced by steroidal nondepolarizing NMBAs [8]. Matsui M et al. reported that 4 mg·kg^− 1^ of sugammadex can provide a complete and rapidly facilitated recovery from rocuronium-induced deep neuromuscular blockade to a TOFR of 0.9 in infants and children [9]. To date, pediatric studies are limited and have focused on using sugammadex to reverse neuromuscular blockade in pediatric patients aged 2–18 years [10, 11].

Currently, sugammadex is approved for children ≥2 years in China after its introduction in April 2017. Therefore, this prospective pilot study aimed to investigate the time between the injection of sugammadex and recovery of the TOFR to ≥0.9 from rocuronium-induced neuromuscular blockade in Chinese children aged 1–12 years and evaluate whether age influences the effect of sugammadex in reversing rocuronium-induced neuromuscular blockade.

## Methods

### Study design

This single-center, prospective, parallel-groups clinical trial was approved by the Ethics Committee of Shanghai Children’s Medical Center in China (SCMCIRB-K2018086) and registered at the Chinese Clinical Trial Registry website on June 8, 2019 (http://www.chictr.org.cn/index.aspx, ChiCTR1900023715). All methods in this study were carried out in accordance with relevant guidelines and regulations. Written informed consent was obtained from the parents or legal guardians of each child before the trial began. The study was conducted at the Shanghai Children’s Medical Center from August 2019 to August 2020.

### Patient selection

The study included children aged 1–12 years who had American Society of Anesthesiologists physical status I or II and were scheduled for lower abdominal surgery under general anesthesia. The exclusion criteria were the presence of a disorder known to affect neuromuscular transmission, recent upper respiratory infection, congenital heart disease, any contraindication for any study drug or scheduled anesthetic drug, and lack of parental/guardian consent to participate.

### Anesthesia procedure

To facilitate venous cannulation of the pediatric patients, 0.5 mg·kg^− 1^ midazolam, with a maximum dosage of 15 mg, was administered orally 30 min before admission to the operating room. Electrocardiography, pulse oximetry (SpO_2_), peripheral temperature, and noninvasive blood pressure were monitored in all patients. Anesthesia was induced via intravenous administration of fentanyl 3 μg·kg^− 1^, propofol 2.0–3.0 mg·kg^− 1^, and rocuronium 0.6 mg·kg^− 1^. After tracheal intubation, patients were ventilated using the pressure-controlled ventilation mode with the following parameters: inspiratory pressure, 10–16 mmHg; respiration rate, 16–22 per min; inspiration time, 1.0–1.2 per s; inhaled gas oxygen concentration (fraction of inspired oxygen), 0.5–0.6; positive end-expiratory pressure, 4–5 mmHg. End-tidal carbon dioxide pressure (ETCO_2_) was maintained at 35–45 mmHg via mechanical ventilation. Anesthesia was maintained with 0.2–0.3 μg·kg^− 1^·min^− 1^ remifentanil infusion and 1.0–1.2 minimal alveolar concentration (MAC) sevoflurane inhalation. If the TOF count became ≥1, additional doses of rocuronium 0.2–0.3 mg·kg^− 1^ were administered intraoperatively. If the heart rate or mean arterial pressure decreased > 20% compared with preoperative values, the remifentanil dose and sevoflurane concentration were reduced accordingly. All patients received dexamethasone 0.1 mg·kg^− 1^ and tropisetron 0.1 mg·kg^− 1^ as anti-emetic. Sodium acetate Ringer’s injection was infused during surgery in accordance with a previously described 4:2:1 weight-based formula [12].

### Neuromuscular monitoring

Neuromuscular function was monitored quantitatively via kinemyography using an anesthesia monitor (GE S/5 AM; GE Healthcare Co., Helsinki, Finland) with electrodes placed at the ulnar nerve of the adductor pollicis muscle. The data obtained were stored in the hospital data system using the GE S/5 AM Monitoring version M1236712–1.0 T4 (GE Healthcare Co.). After supramaximal stimulation (current ≤70 mA) with square-wave pulses for a duration of 0.2 ms, repetitive TOF stimulation of 2 Hz was administered. The baseline TOFR was measured in every patient when unconscious via intravenous administration of propofol 1.5–2.0 mg·kg^− 1^. Rocuronium was administered after the baseline value was obtained. TOF was assessed every 5 min during surgery and every 15 s at the end of the surgery, and subsequent data collection continued.

### Reversal of neuromuscular blockade and extubation

At the end of the surgery, remifentanil infusion was discontinued, and the sevoflurane concentration was adjusted to 0.6 MAC to maintain anesthesia. To achieve more accurate single doses of sugammadex, it was diluted to a final concentration of 10 mg·mL^− 1^ with 0.9% normal saline; subsequently, 2.0 mg·kg^− 1^ sugammadex was administered after TOF count two (TOFC 2) for the reversal of rocuronium-induced neuromuscular blockade. A TOFR ≥0.9 measured at the adductor pollicis muscle was defined as “adequate recovery” from neuromuscular block [2]. Observation was continued for 5 min after the TOFR recovered to 0.9, and the incidence of the TOFR dropping to below 0.9 was recorded.

When the TOFR reached 0.9, sevoflurane inhalation was ceased, and 100% oxygen was administered at a flow rate of 6 L·min^− 1^ to wash out the respiratory circuit. After a TOFR of 0.9 was attained, the standard criteria for awake extubation in children were assessed, including eye-opening, facial grimace, body movement other than coughing, end-tidal concentration of sevoflurane < 0.3%, oxygen saturation > 95%, and tidal volume > 5 mL·kg^− 1^ [13]. After successful extubation, patients were transferred to the post-anesthesia care unit (PACU) for recovery until consciousness and stable vital signs were regained. In addition to vital signs, end-tidal capnography was simultaneously recorded using a handheld Newtech® capnograph/pulse oximeter (NT1D; Shenzhen Newtech, Inc., Guangdong, China), and nasal cannulation for capnography was applied in every patient via the sidestream sampling port. To prevent expiratory gas from being diluted by the air, the patients were asked to keep calm, and the sampling distance was shortened as much as possible and the maximum value of end-tidal capnography at the end of the plateau was recorded. Vital signs and recovery were evaluated every 10 min using the modified Aldrete score until the patient fulfilled the criteria for safe discharge.

### Data collection

Patients who met the inclusion criteria were assigned to one of three age groups: 1 to < 3 years, 3 to < 5 years, or 5 to ≤12 years.

The primary outcome was the time from starting sugammadex administration until the TOFR recovered to 0.9. Secondary outcomes included the number of recurarization patients (time from TOFR recovery to 0.9 until 5 min), extubation time (time from sugammadex administration to tracheal extubation), PACU time (length of stay in the PACU), and complications, which included allergic reaction, postoperative nausea and vomiting (PONV), and bronchospasm. Additional outcomes included measurement of hemodynamic parameters (before and 5 min after sugammadex administration), pulse oxygen saturation, heart rate, systolic blood pressure (SBP), diastolic blood pressure (DBP), and ETCO_2_ in the PACU every 10 min (time from PACU admission until fulfilled criteria for PACU discharge). The surgery time and body core temperature at the end of the surgery were recorded.

### Sample size

According to a previous study [14], the mean (standard deviation [SD]) time from sugammadex administration to TOFR recovery to 0.9 was 1.2 (0.4) min in children. We assumed a difference of 0.2 min for the mean time between injection of sugammadex and recovery of the TOFR to ≥0.9 among the different age groups. Based on these parameters, the power and sample size program utilized in the current study [15] indicated that a minimum sample size of 21 patients per group was required at the 0.05 level of significance to provide 80% power. After factoring in a potential dropout rate of 10%, the required initial sample size was 69 children.

### Statistical analyses

Statistical analysis was performed using IBM SPSS Statistics version 16 (IBM Corp., Armonk, NY, USA). Summary statistics (mean and standard deviation) were derived from continuous variables; frequencies and percentages were derived from categorical variables. The Kolmogorov–Smirnov test was used to verify normal data distribution. Comparisons of continuous data were performed using the paired-samples *t*-test and one-way analysis of variance. Comparison of hemodynamics was performed using two-way repeated measures. The Kruskal–Wallis test was used to compare nonparametric variables. Categorical variables were compared using the chi-square test or Fisher’s exact test if > 20% of cells with an expected count of < 5 were observed. A *p*-value < 0.05 indicated statistical significance.

## Results

**Fig. 1:**
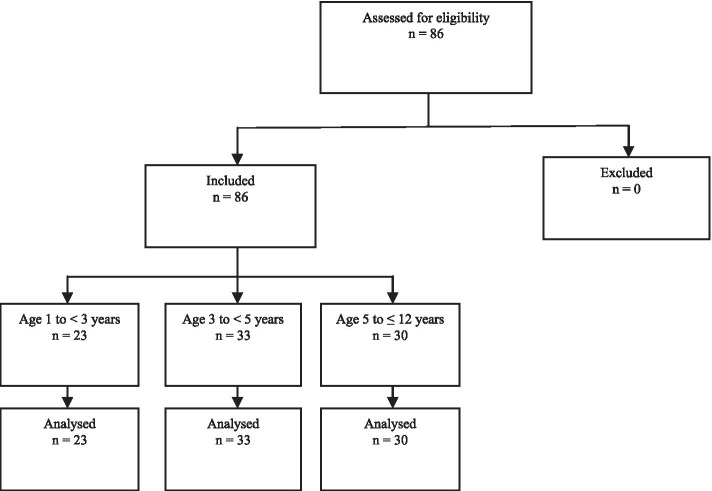
Flow diagram

A total of 86 children were recruited into the trial, including 23 aged 1 to < 3 years, 33 aged 3 to < 5 years, and 30 aged 5 to ≤12 years (Fig. 1 and Table 1).

**Table 1:** Baseline patient characteristics

	Age range			Statistic	*p*-Value
	1 to < 3 years (*n* = 23)	3 to < 5 years (*n* = 33)	5 to ≤12 years (*n* = 30)		
Age (years)	1.9 ± 0.5	3.9 ± 0.5	6.9 ± 1.3	F = 230.76	< 0.001
Sex				*χ*^2^ = 3.783	0.151
Male	15	23	14		
Female	8	10	16		
Weight (kg)	12.8 ± 1.9	17.6 ± 3.5	27.3 ± 7.5	F = 58.268	< 0.001
Height (cm)	86.7 ± 7.1	104.4 ± 5.1	125.0 ± 9.9	F = 167.9	< 0.001
Duration of surgery (min)	24 ± 9.8	26 ± 11.9	26.2 ± 8.7	F = 0.374	0.689
Baseline TOFRs	0.9 ± 0.1	0.9 ± 0.1	0.9 ± 0.1	F = 0.84	0.435

All data except the sex distributions are expressed as means ± standard deviations

The times between 2.0 mg·kg^−1^ sugammadex administration and recovery of the TOF ratio ≥ 0.9 were similar in all three groups (F = 0.691, *p* = 0.504). Extubation time was similar in all three groups (F = 2.097, *p* = 0.129). Differences in length of stay in the PACU were not significant among the groups (F = 0.207, *p* = 0.814) (Table 2). Subsequent TOFR decrease after initial TOFR recovery to 0.9 was not observed in any of the three groups.

**Table 2:** Summary of recovery after sugammadex administration

Variables	1 to < 3 years	3 to < 5 years	5 to ≤12 years	Statistic	*p*-Value
Ta (min)	1.9 ± 0.8	1.7 ± 0.7	1.8 ± 0.6	F = 0.691	0.504
Tb (min)	13.3 ± 4.5	12.5 ± 4.7	11.1 ± 2.4	F = 2.097	0.129
Tc (min)	24.4 ± 11.3	25.2 ± 11.5	23.5 ± 9.5	F = 0.207	0.814
Adverse events					
Rash	0	1	0		1.000
PONV	0	1	0		1.000
Bronchospasm	0	0	0		N/A

Data are expressed as means ± standard deviations. The categorical variables are expressed as numbers

Ta, time between sugammadex administration and recovery of the TOFR to ≥0.9; Tb, extubation time; Tc, PACU time; PONV, postoperative nausea and vomiting

Comparisons of heart rate for “time” and for the interaction “time × age group” were significant among the groups. Heart rate at 5 min after sugammadex administration was significantly lower than that at baseline in children aged 3 to < 5 years and 5 to ≤12 years (Table 3).

**Table 3:** Hemodynamic parameters after sugammadex administration

Variables	Time	1 to < 3 years	3 to < 5 years	5 to ≤12 years	*p-*Value for time	*p-*Value for time×age group
HR (beats/min)	Before sugammadex administration	102.5 ± 14.8	101.6 ± 11.8	99.9 ± 18.7	< 0.001	< 0.001
	5 min after sugammadex administration	97.2 ± 19.6	90.5 ± 17.9	76.6 ± 15.1		
*p*^a^ -Value		0.091	< 0.001	< 0.001		
SBP (mmHg)	Before sugammadex administration	93.6 ± 10.1	96.9 ± 10.1	105 ± 13.3	< 0.001	0.482
	5 min after sugammadex administration	86.1 ± 9.8	87.3 ± 8.6	92.9 ± 8.5		
DBP (mmHg)	Before sugammadex administration	44.8 ± 9.9	53.6 ± 8.5	56.9 ± 10.7	< 0.001	0.180
	5 min after sugammadex administration	42.4 ± 8.3	48.8 ± 9.8	48.1 ± 7.5		

Data are expressed as means ± standard deviations

HR, heart rate; DBP, diastolic blood pressure; SBP, systolic blood pressure

^a^Comparison between before sugammadex administration and 5 min after administration

The mean ETCO_2_ values at the 0-, 10-, 20-, or 30-min time points in the PACU were 52.2 ± 6.7, 48.1 ± 4.3, 46.3 ± 4.0, and 43.3 ± 6.6 mmHg in children aged 1 to < 3 years; 49.9 ± 10, 45.9 ± 7.9, 42.5 ± 6.2, and 41.2 ± 5.3 mmHg in children aged 3 to < 5 years; 49.1 ± 10.2, 46.3 ± 8.2, 42.7 ± 8.0, and 42.7 ± 8.4 mmHg, respectively. There were no significant differences in ETCO_2_ among the three groups at the 0-, 10-, 20-, or 30-min time points in the PACU (*p* > 0.05).

There were no significant differences in the number of adverse events, including rash, PONV and bronchospasm, among the three groups (*p* > 0.05) (Table 2). In patients with adverse events, the time between the injection of sugammadex and recovery of the TOFR to ≥0.9 was not delayed, and there was no recurrence of neuromuscular blockade after the TOFR recovered to > 0.9.

## Discussion

In our prospective pilot study, we did not observe any differences in the effects of sugammadex as a reversal agent on the recovery of TOFR to 0.9 between age groups in children aged 1–12 years. The extubation time after sugammadex administration was similar among the groups. The heart rate at 5 min after sugammadex administration was significantly lower than that at baseline in children aged 3 to < 5 years and 5 to ≤12 years.

The mean times between 2.0 mg·kg^− 1^ sugammadex administration and recovery of the TOF ratio ≥ 0.9 were similar in all the groups, which is concordant with previous reports [14]. The incidence of TOFR decreasing to < 0.9 after it had been reversed to ≥0.9 via sugammadex administration (2.0 mg·kg^− 1^) was not observed in any of the patients aged 1–12 years. A previous study reported that no difference in recovery was observed between the use of 2.0 mg·kg^− 1^ and 4.0 mg·kg^− 1^ sugammadex to reverse rocuronium-induced deep neuromuscular blockade from the re-appearance of post-tetanic count to a TOFR of 0.9 in infants and children [9]. This indicates that 2.0 mg·kg^− 1^ sugammadex may be sufficient to fully reverse rocuronium-induced moderate neuromuscular blockade in pediatric patients aged 1–12 years. Both rocuronium and sugammadex are water-soluble and easily distributed in extracellular fluids; the percentage of extracellular fluids in young children is higher than that in older children [16]. However, the distribution and redistribution of rocuronium and sugammadex in extracellular fluids and the neuromuscular junction do not influence the rates of rocuronium-induced neuromuscular blockade recurrence in pediatric patients aged 1–12 years after 2.0 mg·kg^− 1^ sugammadex administration [7].

The heart rate in pediatric patients aged 3 to ≤12 years was significantly reduced after sugammadex administration under 0.6 MAC sevoflurane inhalation anesthesia. These results are consistent with those of a previously reported study in children with congenital heart disease undergoing a cardiac operation, in which the heart rate was significantly lower after sugammadex administration than before administration [17]. In that study, except for sugammadex administration, other factors, such as type of congenital heart disease, body temperature, and operation technique, may influence heart rate and interfere with the judgment of the relationship between sugammadex administration and vital signs. Contrastingly, children undergoing non-cardiac surgery with sevoflurane inhalation were focused on in our study. To the best of our knowledge, heart rate increased during 1 and 2 MAC of sevoflurane anesthesia in pediatric patients [18]. However, it seemed that 0.6 MAC sevoflurane could not antagonize the effects of sugammadex administration on bradycardia.

The extubation time after sugammadex administration was similar in all the patients aged 1 to ≤12 years. Sugammadex administration resulting in faster extubation times than neostigmine in children aged 2–10 years has been reported in a previous study [3]. Further, in the current study, extubation times associated with sugammadex administration under sevoflurane maintenance were compared in children of different age groups. In addition to RNMB, other factors, such as midazolam premedication, residual inhalation anesthetics, hypercapnia, and hypothermia associated with successful extubation, were considered in the present study. Clinical muscle function observations, including conjugate gaze, facial grimace, eye-opening, purposeful movement, and tidal volume > 5 mg·kg^− 1^ were also performed to confirm the suitability of extubation. Therefore, multiple factors influence the extubation process, and no difference in extubation time with sugammadex was found in children aged 1 to ≤12 years in this cohort.

In the current study, ETCO_2_ was in the normal range while the patients stayed in the PACU, and there were no significant differences in ETCO_2_ levels among the three age groups. These results are consistent with those of a previous study, in which no airway-related complications occurred in pediatric patients during the recovery from inhalational anesthetics [19]. In that study, the no-touch technique was adopted as an intervention related to airway and ventilator management, and there was no consideration of RNMB. To the best of our knowledge, RNMB is a major risk factor for respiratory insufficiency in children, particularly in the early stages after surgery, during which RNMB is associated with worsening of the ventilator response to hypoxia and predisposition to respiratory complications [7]. Therefore, by precluding RNMB, sugammadex can prevent post-operative airway obstruction and decrease the occurrence of NMBA-induced ventilator drive decrease equivalently in children aged 1–12 years.

After sugammadex administration, a 4-year-old boy vomited once and a 3-year-old boy developed a rash, which disappeared gradually 30 min later without anti-anaphylaxis treatment. These self-limiting issues appeared to be well-tolerated by the children, as reported in a previous study [10].

Our study had some limitations. First, it was conducted at a single center. Multicenter studies with larger collective sample sizes are necessary to further investigate the significant associations detected in this study. Second, the dosages of sugammadex required to achieve the reversal of neuromuscular blockade in pediatric patients were not investigated. Third, the results do not constitute a complete evaluation of the safety and efficacy of sugammadex in pediatric patients because it is not used in children aged < 1 year at our hospital. Thus, the inclusion of children aged < 1 year should be considered in future studies. Fourth, sevoflurane can increase the degree of muscle relaxation and prolong recovery caused by rocuronium and may thus potentially interfere with the clinical efficacy of sugammadex during the recovery phase.

## Conclusions

We concluded that Chinese children’s age has no influence on sugammadex reversal of rocuronium-induced moderate neuromuscular blockade. However, the decrease in hemodynamic parameters after sugammadex administration requires further attention.

## Data Availability

The datasets used and/or analyzed during the current study are available from the corresponding author upon reasonable request.

## Abbreviations

DBP: diastolic blood pressure
ETCO_2_: end-tidal carbon dioxide pressure
HR: heart rate
MAC: minimal alveolar concentration
NMBAs: neuromuscular blocking agents
PACU: post-anesthesia care unit
PONV: postoperative nausea and vomiting
RNMB: residual neuromuscular blockade
SBP: systolic blood pressure
SD: standard deviation
SpO_2_: pulse oximetry
TOF: train-of-four
TOFR: train-of-four ratio
